# Beyond the Cell Surface: Targeting Intracellular Negative Regulators to Enhance T cell Anti-Tumor Activity

**DOI:** 10.3390/ijms20235821

**Published:** 2019-11-20

**Authors:** Poojitha Sitaram, Bradley Uyemura, Subramaniam Malarkannan, Matthew J. Riese

**Affiliations:** 1 Department of Surgery, Medical College of Wisconsin, Milwaukee, WI 53226, USA; psitaram@mcw.edu; 2 Blood Research Institute, Blood Center of Wisconsin, Versiti, Inc, Milwaukee, WI 53226, USA; SMalarkannan@Versiti.org; 3 Department of Medicine, Division of Hematology/Oncology, Medical College of Wisconsin, Milwaukee, WI 53226, USA; buyemura@mcw.edu; 4 Department of Microbiology and Immunology, Medical College of Wisconsin, Milwaukee, WI 53226, USA

**Keywords:** T lymphocyte, E3 ubiquitin ligases, phosphatases, kinases

## Abstract

It is well established that extracellular proteins that negatively regulate T cell function, such as Cytotoxic T-Lymphocyte-Associated protein 4 (CTLA-4) and Programmed Cell Death protein 1 (PD-1), can be effectively targeted to enhance cancer immunotherapies and Chimeric Antigen Receptor T cells (CAR-T cells). Intracellular proteins that inhibit T cell receptor (TCR) signal transduction, though less well studied, are also potentially useful therapeutic targets to enhance T cell activity against tumor. Four major classes of enzymes that attenuate TCR signaling include E3 ubiquitin kinases such as the Casitas B-lineage lymphoma proteins (Cbl-b and c-Cbl), and Itchy (Itch), inhibitory tyrosine phosphatases, such as Src homology region 2 domain-containing phosphatases (SHP-1 and SHP-2), inhibitory protein kinases, such as C-terminal Src kinase (Csk), and inhibitory lipid kinases such as Src homology 2 (SH2) domain-containing inositol polyphosphate 5-phosphatase (SHIP) and Diacylglycerol kinases (DGKs). This review describes the mechanism of action of eighteen intracellular inhibitory regulatory proteins in T cells within these four classes, and assesses their potential value as clinical targets to enhance the anti-tumor activity of endogenous T cells and CAR-T cells.

## 1. Introduction

T cells play a central role in mediating adaptive cellular immune responses, including those directed against tumor cells. T cells are subject to numerous physiological restraints that prevent inappropriate activation by self-peptide or sustained activation and subsequent development of autoimmunity. The primary mechanism of eliminating self-reactive T cells occurs during thymic development in which T cells with overly vigorous binding to self-peptide are clonally deleted through negative selection prior to exit from the thymus. An additional safeguard to prevent self-reactivity occurs when T cells that bind to peptide-MHC (major histocompatibility complex) in the periphery in the absence of costimulation undergo inactivation by a process termed anergy. More recently, expression of inhibitory cell surface receptors on T cells have been identified as playing an essential role in acting as “checkpoints” to limit T cell activity. These receptors can be broadly classified into two categories, (i) proteins important in attenuating early activation events, best characterized by CTLA-4 (which restricts optimal costimulation by ligands for Cluster of Differentiation 28 (CD28)), and (ii) proteins upregulated after chronic exposure of T cells to cognate antigen, such as PD-1. Both these classes of receptors have demonstrated the capacity to inhibit CD8^+^ T cell response to tumor, and antibodies that block either of these receptors have been approved by the U.S. Food and Drug Administration for the treatment of patients with cancer. Apart from PD-1, other inhibitory checkpoint receptors (ICR) that are upregulated in “exhausted” CD8^+^ T cells after chronic antigen exposure include T cell immunoglobulin and mucin-domain containing-3 (Tim3), Lymphocyte-activation gene 3 (Lag-3), and Natural Killer Cell Receptor 2B4. While single and combination therapies targeting ICRs have demonstrated impressive responses in many instances, a majority of patients remain unresponsive, relapse frequently occurs (with the notable exception of CTLA-4-responsive disease), and vigilance for induction of autoimmunity is required.

Apart from immune checkpoint receptors, numerous other inhibitory ligands for CD8^+^ T cells are abundantly present within the tumor microenvironment. These include adenosine, prostaglandin E_2_, and the pleiotropic cytokine Transforming growth factor beta (TGFβ). Pharmaceutical agents that manipulate the activity of all three of these ligands are currently in development, however, targeting each has posed challenges, with questionable single-agent activity with adenosine and prostaglandin E_2_, and significant off-tumor effects with TGFβ. Thus, there is significant interest in identifying novel targets capable of potently and specifically improving activity of intratumoral CD8^+^ T cells.

Recently, our group and others have attempted to define the value of targeting intracellular checkpoint proteins (ICPs) to enhance CD8^+^ T cell anti-tumor activity. ICPs are negative regulators of signal transduction downstream of the T cell receptor (TCR) that facilitate termination of TCR signaling. ICPs fall into one of two broad categories: ubiquitin E3 ligases, which target TCR signaling mediators for degradation by the proteasome, and signaling kinases/phosphatases, which directly antagonize TCR-mediated signaling through de-activation of signaling mediators or activation of antagonistic proteins. ICPs represent potentially superior targets to ICRs since they can simultaneously confer insensitivity to multiple inhibitory cell surface receptors. This review will compare and contrast the mechanism of action and anti-tumor activity of ICPs in CD8^+^ T cells.

## 2. E3 Ubiquitin Ligases that Negatively Regulate T Cells

Ubiquitination is the process of attaching ubiquitin, a highly conserved 76 amino acid protein moiety to substrate proteins, thereby targeting them for degradation in the proteasome or endosome. Cells use this process as a mechanism to eliminate misfolded or unwanted proteins and to regulate levels of transcription factors and mediators of signal transduction. Ubiquitination takes place in three enzymatic steps, two of which are substrate agnostic, and a final step which requires recognition of the protein substrate targeted for degradation. First, an E1 ubiquitin-activating enzyme binds ubiquitin through ATP hydrolysis. Next, an E2 ubiquitin-conjugating enzyme catalyzes the transfer of the ubiquitin from the E1 to an activated state within the E2 molecule itself. Finally, an E3 ubiquitin ligase, which contains the substrate recognition component of the ubiquitination process, brings the E2 in contact with the targeted substrate and mediates the transfer of the ubiquitin from the E2 to a lysine residue on the substrate protein. Mammals have only one reported E1 enzyme, which binds over 30 E2 enzymes, which in turn can bind to hundreds of E3 ligases [1,2]. Substrate specificity is therefore maintained by the E3 ubiquitin ligase in this process.

**Figure 1:**
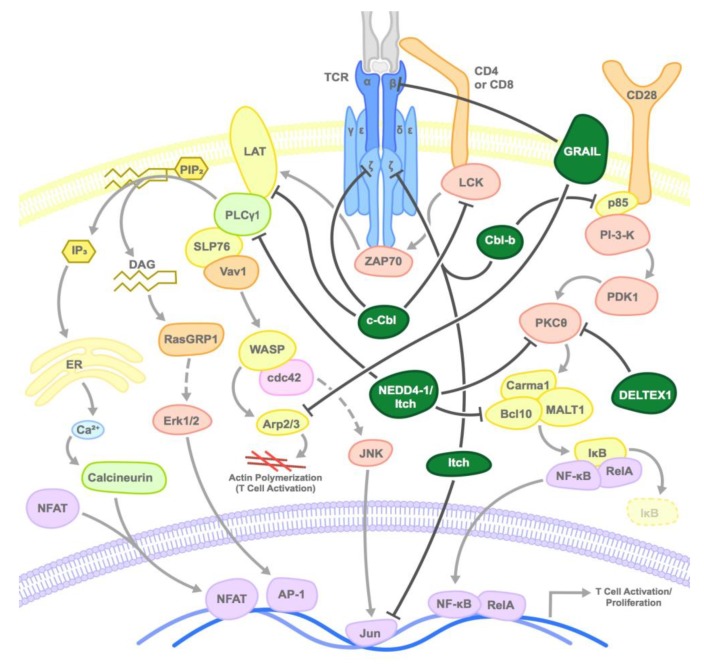
Mode of inhibition of T cell activation by E3 ubiquitin ligases: E3 ubiquitin ligases inhibit T cell receptor (TCR) activation at various steps by inducing the proteasomal degradation or sequestration of various substrates as shown. Dark green circles represent the E3 ligases and arrows point to primary target substrates.

Post-translational modification of proteins by ubiquitination plays an important role in multiple cellular processes such as regulation of the cell cycle, cell differentiation, gene transcription, DNA repair, and in the modulation of immune responses. In the innate immune system, antigen presentation depends on the ubiquitin-mediated degradation of foreign proteins [3]. Moreover, the Skp1-Cullin1-F-box (SCF) RING (Really Interesting New Gene) E3 ubiquitin ligases are critical regulators of T cell function by inducing the activation of Nuclear factor kappa-light-chain-enhancer of activated B cells (NF-κB, a sine quo non pro-inflammatory transcription factor) by the ubiquitin-mediated degradation of I-κB (Inhibitor of κB) inhibitory protein, which releases NF-κB, allowing nuclear translocation and transcriptional activation [4]. In the following section, we will focus on the function of E3 ubiquitin ligases that negatively regulate T cell function (Figure 1).

### 2.1. CBL Family

The Cbl (Casitas B-lymphoma) proteins are a highly conserved family of proteins with three isoforms–c-Cbl, Cbl-b, and Cbl-3 [5,6,7]. The domain structure of these proteins is highly conserved with each possessing an N-terminal tyrosine kinase binding (TKB) domain, a proline-rich protein-protein interaction domain, and a RING finger (RF) catalytic domain which is responsible for the ubiquitin transfer activity of these proteins [8]. While the physiological function of Cbl-3 has yet to be fully defined, the other two family members, Cbl-b and c-Cbl, have both been shown to act as negative regulators of TCR signaling [9,10].

C-Cbl, the first identified member of this protein family, is predominantly expressed in brain, immune, and reproductive cells but its highest level of expression and function is found in the thymus [6,11,12]. C-Cbl binds and targets numerous T cell proteins for degradation, however, its primary activity in T cells is in targeting the zeta chain of the T cell receptor complex for degradation after TCR activation, thereby leading to downregulation of TCR signaling [13]. In this role, immature c-Cbl deficient T cells have increased expression of TCR-CD3ζ complexes and undergo more efficient positive selection [14]. The interaction between c-Cbl and ζ chain is facilitated by the ζ-chain-associated protein tyrosine kinase, Zeta-chain-assocyated protein kinase 70 (Zap-70), a critical tyrosine kinase that helps initiate TCR signaling and which also contributes to termination of TCR signaling by acting as a scaffold between c-Cbl and the CD3ζ chain [13]. Intriguingly, evidence suggests that ubiquitination of some cell surface receptors by c-Cbl can result in their translocation into vesicles for degradation in the lysosome, independent of the proteasome, and this may be the mechanism of CD3ζ degradation by Cbl-b [15,16]. Two additional important targets for c-Cbl-mediated degradation in activated T cells include the proximal activating cytoplasmic tyrosine kinase Lck (lymphocyte-specific protein tyrosine kinase) and the adaptor protein LAT (linker for activation of T cell), both of which are required for TCR signal transduction [17]. Depletion of c-Cbl results in a defect in TCR-mediated LAT internalization and elevated levels of LAT within T cells [18]. c-Cbl has also been linked with T cell anergy via its interaction with the CrkL (Crk like) adaptor protein and C3G, the guanine nucleotide-exchange factor for Rap1 (Ras-proximate-1). Rap1 is known to contribute to the T cell anergic state as increased Rap1 activation has been observed in anergic T cells [19], however, this may not be physiologically relevant since lack of c-Cbl has been demonstrated to impair, not enhance, establishment of T cell anergy [20,21].

In contrast to c-Cbl, Cbl-b is ubiquitously expressed, but has higher expression and function in peripheral T-cells compared with other tissues [22]. While c-Cbl predominantly acts through degradation of components of the TCR complex itself, Cbl-b is thought to function largely by regulating T cell activity through degradation of phospho-inositol-3-kinase (PI-3-K) downstream of the CD28 costimulatory receptor. In the absence of Cbl-b, naïve T cell activation and cytokine production can proceed independently of CD28 costimulation [23,24]. Important substrates of PI-3-K during T cell activation include Vav and NF-κB [24]. Vav, a guanine exchange factor in the Rho GTP hydrolase (GTPase) family, helps facilitate T cell activation via TCR clustering in a CD28-dependent manner, which may also be dependent on PI-3-K [25,26,27]. Cbl-b acts to attenuate PI-3-K signaling through ubiquitination of p85, the regulatory subunit of PI-3-K, thereby interfering with its ability to activate PKCθ and other pathways. In a negative feedback cycle, activation of CD28 leads to Cbl-b ubiquitination and degradation by the 26S proteosome via phosphorylation by protein kinase C theta (PKCθ) [28,29]. Phosphorylation of Cbl-b by PKCθ leads to Cbl-b ubiquitination, facilitated either by Cbl-b itself or by other E3 ligases, of which the HECT (homologous to the E6-AP carboxyl terminus) E3 ligase Nedd (neural precursor cell expressed developmentally down-regulated protein) is the prime candidate [28,30]. Additionally, it has been shown that binding of the CD28 ligands B7-1 or B7-2 (CD80 or CD86) to CTLA-4 on T cells results in an increase in protein level of Cbl-b, suggesting that Cbl-b might also contribute to the inhibitory function of CTLA-4 [31].

Although targeting Cbl proteins may result in an increased potential for T cell-mediated autoimmunity, this concern is contrasted with an enhanced capacity to eliminate pathogens, such as Lymphocytic choriomeningitis virus (LCMV) or fungi [32,33], or enhanced anti-tumor immunity. Cbl-b deficient mice can efficiently reject transplanted tumor xenografts, ultraviolet-light-induced skin tumors, and spontaneously generated T-cell lymphomas in an ataxia telangiectasia mutated (ATM) deficient background [24,34,35]. In this context, immunotherapies incorporating Cbl-b deficiencies could have clinical utility. Spontaneous rejection of TC-1 (tissue culture number one) tumors was observed with the adoptive transfer of naïve polyclonal CD8^+^ Cbl-b-/- T cells into *rag2* (recombination activating gene 2) mutant mice [35]. Cbl-b deficient T cells have also been shown to be less susceptible to immune suppression by regulatory T cells (Tregs), TGFβ and programmed death-ligand 1 (PD-L1) [34,35,36]. Additionally, the combination of therapies targeting CTLA-4 (but not PD-L1) with Cbl-b deficient T cells acts synergistically to enhance anti-tumor response and survival in melanoma mouse models when compared to each of these therapies individually [36], further suggesting that Cbls may be useful clinical targets. A phase I study is completed and an additional phase I study is underway evaluating APN401 (peripheral blood mononuclear cells transfected with siRNA against Cbl-b; Table 1) [37]. Moreover, small molecular inhibitors against Cbl-b are in development [38].

### 2.2. GRAIL

Gene related to anergy in lymphocytes (GRAIL or RNF128) is another RING E3 ubiquitin ligase that has been demonstrated to be an essential enforcer of T cell tolerance. GRAIL was initially identified with Cbl-b as a protein upregulated in anergic T cells relative to resting or activated T cells [41]. It is unique as an E3 ligase because it is a type I transmembrane protein as opposed to a cytosolic protein. GRAIL localizes to a transferrin-recycling endocytic pathway in T cells and endocytosis is required for the implementation of its inhibitory function. This suggests that the targets for GRAIL-mediated ubiquitination and degradation may be endocytosed substrates. Overexpression of GRAIL inhibits the expression of IL2 and IL4 production and induces anergy in CD4+ T cells. GRAIL-mediated anergy can be reversed by expression of a dominant-negative form of GRAIL that contains a mutation in its RING domain that obliterates enzymatic function, indicating that GRAIL activity in CD4+ T cells is dependent on its E3 ligase activity [42]. Mice deficient in GRAIL demonstrate resistance to induction of T cell anergy, TCR-mediated hyperactivation of CD4+ T cells, and increased proliferation and survival of T cells after activation [43,44]. GRAIL-deficient mice also show increased susceptibility to autoimmune diseases [44].

Multiple targets of GRAIL that could impact T cell activation have been identified. GRAIL deficient mice show enhanced ERK1/2 (extracellular signal-regulated protein kinases ½) phosphorylation after T cell activation, indicating that GRAIL regulates proteins upstream from this signaling node [45]. Markedly increased levels of components of the TCR receptor complex are present in GRAIL-deficient CD4+ T cells, including TCRβ and CD3ζ [44] which may result from direct targeting of CD3ζ by GRAIL, since CD3ζ ubiquitination is greatly diminished in GRAIL deficient mice. Alternatively, GRAIL may function by targeting TCRβ since overexpression of GRAIL is sufficient to induce TCRβ downmodulation in CD4+ T cells. Other groups have identified other potential targets of GRAIL including CD40, CD83 and tetraspanins such as CD81 and CD151 [46,47,48]. CD83 has been demonstrated to contribute to costimulation in CD4+ T cells, and CD40 is important in permitting dendritic cell-mediated licensing of T cells [49,50]. GRAIL also ubiquitinates proteins involved in the regulation of the actin cytoskeleton such as Rho guanine dissociation inhibitor (RhoGDI), actin related protein 2/3 (Arp2/3) subunit 5 and coronin 1A, which may play a role in modulating the anergic T cell state [51,52].

More recently, GRAIL has been evaluated in the context of tumor-infiltrating lymphocytes. GRAIL, but not Cbl-b, is upregulated CD8^+^ T cells present within transplanted EL-4 and EG-7 (EL4-ova) lymphoma tumors [53]. Thus, GRAIL may be an important T cell-intrinsic factor that attenuates CD8^+^ T cell responses within tumor. The increased effector function of CD8^+^ T cells in the absence of GRAIL could be in part related to increased expression of interleukin 21 receptor (IL-21R), which has been shown to be an additional direct substrate of GRAIL E3 ligase activity [53], leading to increased IL-21R signaling and release of cytokines via activation of the janus kinase/signal transducer and activator of transcription (JAK-STAT) pathway by enhanced phosphorylation of Jak1, STAT1, STAT3 and STAT5. In this same study, GRAIL was found to be increased and IL-21R decreased in PBMCs isolated from patients with lymphoma when compared with healthy donors, suggesting that targeting GRAIL clinically (most likely via a small molecule inhibitor) may be especially useful in the treatment of lymphoma.

### 2.3. NEDD4 Family

There are 9 members of the NEDD4 (Neural Precursor Cell Expressed, Developmentally Down-Regulated 4) family HECT E3 ubiquitin ligases. NEDD4 family E3 ligases are distinct from Cbl E3 ligases, in that they utilize a HECT domain to transfer ubiquitin as opposed to a RING domain. Of these, the founding member, NEDD4-1, and Itch have been described to play a role in the regulation of the immune system. The most well studied function of NEDD4-1 is in carcinogenesis as it regulates various oncogenes and tumor suppressors such as phosphatase and tensin homolog (PTEN), c-Myc and RAS [54]. NEDD4-1 is overexpressed in multiple types of human cancers, such as gastric, colorectal, breast and lung cancers, and inhibition of NEDD4-1 can result in significant reduction of tumor growth. In T cells, NEDD4-1 and Itch play a role in the negative feedback regulation of T cell receptor activation by the ubiquitination and degradation of Bcl10 [55]. As part of the MALT1-CARMA1-Bcl10 complex, Bcl10 is a critical activator of the pro-inflammatory transcription factor NF-κB in T cells. Upon TCR/CD28 co-activation, phospholipase C, gamma 1 (PLC-γ1) generates the second messenger diacylglycerol (DAG) which activates PKCθ, which leads to the activation of IκB kinase (IKK) through a MALT1-CARMA1-Bcl10-dependent pathway [56]. Activated IKK phosphorylates IκB inducing its ubiquitination and degradation, thereby removing cytosolic sequestration of NF-κB, and leading to nuclear translocation of NF-κB and institution of its transcriptional program, which includes pro-inflammatory cytokines. Thus, NEDD4-1 and Itch HECT ubiquitin ligases serve as negative regulators by impeding Bcl10-mediated activation of NFκB signaling [55].

The Itch protein was initially identified as part of mutational studies of the mouse coat color gene agouti [57]. Apart from changes in coat color, homozygous mutations in *Itch* result in urticaria, from which its name derives, and susceptibility to spontaneous autoimmunity [58]. Itch has been shown to play a role in tumorigenesis mainly by regulating the Hedgehog and Hippo pathways [54,59,60,61]. Itch also plays an extensive role in regulating the immune response. Itch regulates NF-κB activation in conjunction with NEDD4-1, and when phosphorylated by c-Jun N-terminal kinase (JNK), Itch induces the ubiquitination and proteosomal degradation of c-Jun and JunB [62,63,64]. JunB and c-Jun transcription factors play a role in T helper type 2 (Th2) differentiation, and the depletion of Itch from T cells increases Th2 differentiation after activation. Loss of Itch also results in modest increases in T cell proliferation and interleukin 2 (IL-2) production, but significantly enhanced IL-4 production in Th2 cells. Independent of effects on Th2 differentiation and cytokine production, Itch inhibits the production of IL-17 in the colon mucosa from Th17 CD4+ T cells and innate lymphoid cell subsets such as γδ T cells [65]. These changes likely result from Itch targeting of ROR-γt (RAR-related orphan receptor γt), the essential transcription factor for IL-17 production, for ubiquitination and degradation [65]. Itch may also play a role in Treg CD4+ T cell activity, perhaps through targeting Smad2 [65,66].

Like Cbl-b and GRAIL, Itch is also important for helping mediate T cell anergy. Expression levels of Itch, Cbl-b and GRAIL are increased after induction of calcium-mediated signaling in the absence of AP-1 formation during in vitro induction of T cell anergy, for instance with stimulation of T cells with the Ca2+ ionophore ionomycin. In this process, Itch and NEDD4-1 induce the ubiquitination and degradation of critical signaling proteins downstream of TCR activation, PKCθ and PLC-γ1, leading to the reduced activation of AP-1 [67]. Itch has also been shown to cooperate with other E3 ligases to attenuate immune responses. Double knockout mice missing Itch in combination with either WWP2 (another NEDD4 family member) or Cbl-b exhibit stronger autoimmunity phenotypes that mice deficient in either gene alone [68,69]. In fact, Itch and Cbl-b were found to directly interact to enhance ubiquitination of CD3ζ to terminate TCR signaling.

Itch has also been pursued as a target for cancer therapy; however, the primary focus has been on targeting Itch in tumor cells directly and not necessarily as a means to augment tumor anti-immune response. For instance, small molecule inhibitors of Itch have been pursued as a means to potentiate chemotherapeutics or to induce apoptosis in chronic lymphocytic leukemia [70,71]. It is currently unclear whether targeting Itch will be a useful strategy for enhancing anti-tumor activity. While alterations in T cell function appear most strongly related to enhancement of Th2 CD4+ T cell differentiation in Itch-deficient mice, the similar capacity of Itch with GRAIL and Cbl-b to enforce anergy induction in other T cells warrants further evaluation. Importantly, like other negative regulators of T cell activation, inhibition of Itch can result in potentially deleterious effects. For instance, a case study has been reported of a 1-year old patient that developed multisystem autoimmune disease including autoimmune hepatitis after liver transplant as a result of having a homozygous mutation in *Itch* [72]. It is therefore crucial to remain vigilant for potential autoimmune effects while targeting all immune-relevant E3 ligases, including Itch.

### 2.4. Deltex1

Deltex1, an E3 RING finger ubiquitin ligase expressed upon activation of Notch signaling, is upregulated during T-cell anergy and downregulated after T cell activation [73,74,75]. Downregulation of Deltex1 has been shown to enhance T cell cytokine production [75]. There is some controversy in how Deltex1 negatively regulates T cell activation. Whereas overexpression of Deltex1 in EL4 T cells does not alter levels of PKCθ [75], treatment of Jurkat T cells with a calcium ionophore results in downregulation of PKCθ that can be reversed by decreased expression of Deltex1 [76]. Moreover, PKCθ can be immunoprecipitated with Deltex1 after overexpression of both proteins in 293T cells [76]. The importance of Deltex1-mediated suppression of T cell activation is unclear since deletion of the RING finger domain of Deltex1 only modestly affects its capacity to inhibit T cell function [75]. It is possible that Deltex1 may function through stabilization of Cbl-b in T cells [75]. Mice lacking Deltex1 showed an increase in total mature peripheral lymphocytes, enhanced T cell activation and proliferation, reduced T cell tolerance, and increased susceptibility to autoimmune diseases [74]. The increased immune response in Deltex1 deficient mice suggests that targeting this protein would be potentially beneficial. However, a means to target this protein, especially if the enzymatic activity of the protein is not essential, is unclear.

### 2.5. Other E3 Ligases

In addition to the E3 ligases described in this review, several other proteins have been shown to negatively regulate T cell activation by ubiquitination. TNF receptor-associated factor 6 (TRAF6), an activator of NF-κB, was found to suppress T cell activation by attenuating PI3K-Akt signaling [77]. TRAF6 does not ubiquitinate substrates to target them for degradation, but instead uses ubiquitination to alter substrate protein location or function [78]. Mice with T cell-specific deletion of TRAF6 deficient mice showed a marked increase in activated and effector CD4+ T cells and exhibited splenomegaly and lymphadenopathy, along with the development of systemic inflammation. However, the role of TRAF6 may be pro-inflammatory in some circumstances since a small molecule inhibitor, C25-140, that disrupts binding of TRAF6 to its partner E2 ligase Ubc13, results in decreased TCR-induced activation of NF-κB and decreased psoriasis and rheumatoid arthritis in murine models [79].

Mouse double minute 2 homolog (MDM2), best known as the E3 ligase that ubiquitinates and degrades the tumor-suppressor p53, also targets the T cell activation factor NFATc2 (nuclear factor of activated T cells, cytoplasmic 2) for proteasomal degradation after MDM2 is stabilized by the deubiquitinating enzyme USP15. These enzymatic events may result in attenuation of TCR activity [80,81]. Small molecule inhibitors for MDM2 are under development as a means to enhance p53 activity in tumors that overexpress MDM2. Among various pharmacologic agents, idasanutlin is furthest along in development in a phase III trial in combination with cytarabine in the treatment of refractory AML [82].

The Peli1 RING E3 ligase negatively regulates T cell activation by inducing the ubiquitination and degradation of c-Rel, a member of the NF-κB family. As predicted from its biochemical function, Peli1-deficient mice develop autoimmunity resulting from hyperactivated T cells that are insensitive to suppression by regulatory T cells or TGFβ [83]. Others have demonstrated that NIK is the primary target of Peli1 that regulates NF-κB activation in B cells and shown that B cell-specific loss of Peli1 in mice results in auto-antibody production and lupus [84].

Suppressor of cytokine signaling (SOCS) proteins act as adaptor proteins by binding substrates to the multi-protein RING E3 ligase CBC (Cullin5-ElonginB/C-Rbx2) complex. Eight SOCS proteins have currently been identified, SOCS1-7 and Cish (cytokine-inducible SH2-containing protein). SOCS proteins function as negative regulators of the JAK-STAT pathway mediating the degradation of the JAKs and STATs [85,86,87]. SOCS3 is downregulated in response to T cell activation, and depletion of SOCS3 increases T cell proliferation and the production of IL2 [88]. SOCS6 has been postulated to function as a negative regulator of TCR signaling by targeting the proximal signaling kinase Lck for degradation [89]. Cish also negatively regulates TCR signaling through degradation of PLCγ1 and represents a potential target for enhancing T cell anti-tumor responses [90].

In summary, E3 ubiquitin ligases represent a family of enzymes that regulate the degradation and function of molecules important in T cell activity. It is likely that some of these intracellular immune checkpoints can be targeted to enhance T cell activity in cancer.

## 3. Kinases and Phosphatases that Negatively Regulate T Cells

**Figure 2:**
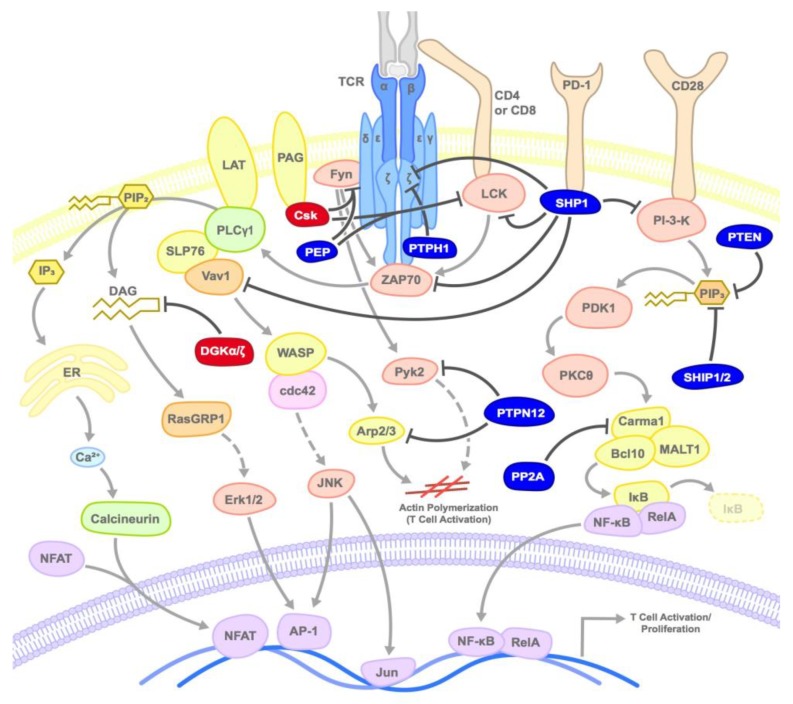
Mode of inhibition of T cell activation by kinases and phosphatases: Kinases or phosphatases inhibit TCR activation at various steps by inducing the phosphorylation or dephosphorylation of various substrates as shown. Dark red circles represent kinases, dark blue circles represent phosphatases, and arrows point to primary target substrates.

The post-translational modification of proteins or lipids by the addition or removal of a phosphate group is a fundamental component of cellular signal transduction. Downstream of the TCR, the activation of proximal tyrosine kinases such as Lck and Zap70 are required for signal transduction and several serine/threonine kinases and lipid kinases are important in modulating signaling activity [91]. In general, most protein phosphorylation events, such as phosphorylation of immunoreceptor tyrosine-based activation motifs (ITAM), result in protein activation or the ability to facilitate protein-protein interaction. In contrast, most dephosphorylation of signaling proteins result in abrogation of activity. Thus, in the context of targeting intracellular immune checkpoints to enhance CD8^+^ T cell anti-tumor activity, most attention has been focused on targeting inhibitory phosphatases. Distinct from kinases and phosphatases that target protein substrates, phosphorylation of lipids can be either stimulatory (e.g., those mediated by PI-3-kinase) or inhibitory (e.g., those mediated by diacylglycerol kinases (DGKs), thus both kinases and phosphatases are potential targets for enhancing immune tumor responses. In this section, we will discuss the roles of phosphatases and kinases that negatively regulate TCR activation, and their potential for clinical targeting as cancer immunotherapeutics (Figure 2).

### 3.1. SHP1/2

SHP1 and SHP2 are Src homology region 2 (SH2) domain-containing protein tyrosine phosphatases (PTPs). SHP1 (*PTPN6*) is expressed predominantly in hematopoietic cells, whereas SHP2 (*PTPN11*) is expressed ubiquitously [91]. The SH2 domains of these proteins play a role in their localization and activity. In the unbound state, the SH2 domain represses the phosphatase activity of these proteins. Once the SH2 domain is bound to substrate, the enzymatic domains of SHPs become accessible leading to dephosphorylation of targets [92,93]. SHP1 and SHP2 are both well-established regulators of TCR signaling, although the exact mechanism by which they perform this function has yet to be fully determined. Potential substrates of SHP1 binding and tyrosine dephosphorylation include Zap-70, Syk, PI3K, Vav, Lck, CD3ζ, and SLP-76, but due to variation in the cell lines used and mode of cellular activation, the precise mechanism of SHP1 function during T cell activation is not yet definitive [94,95,96,97,98,99,100,101]. A relatively novel hypothesis has suggested that SHP1 may also function, in part, by regulating other negative TCR signaling regulators, as SHP1 has been demonstrated to inhibit T cell activation by dephosphorylating Cbl-b, thus protecting it from subsequent ubiquitination and degradation [102]. Independent of considerations of its molecular activity, SHP1 has conclusively been found to negatively regulate T cell activation [103,104]. For instance, deletion of SHP1 allows CD8^+^ T cells to form more stable and longer-lasting bonds with antigen-presenting cells [105,106]. Additionally, mice deficient in SHP1 show increased phosphorylation and activation of Lck, Fyn, and other key components of TCR signaling [107,108], and mice with spontaneous disabling mutations in SHP1 (*motheaten* (*me*) mice) die from autoimmune pneumonitis by three weeks after T cells begin to develop [109,110]. T cells from SHP1 deficient mice also demonstrate hyper-responsiveness to TCR/CD3 stimulation and increased IL2 production. Consistent with these findings, overexpression of SHP1 in T cell lines leads to decreased phosphorylation of TCRζ and LAT, loss of Zap-70 binding to the TCR and reduced IL2 production [111,112].

In contrast to the acceptance of SHP1 as a negative regulator of T cell activation, SHP2 has been demonstrated to function both in positive and negative regulatory capacities in T cells. Earlier studies demonstrated that mice with T cell-specific deletion of SHP2 demonstrate decreased TCR-mediated IL2 production and upregulation of the activation marker CD69 [113]. However, more recent studies have demonstrated that SHP2 can function as a negative regulator when sequestered by and bound to PD1 [114,115,116,117]. Both SHP1 and SHP2 are capable of recruitment to the cytoplasmic tail of PD1 by binding to its immunoreceptor tyrosine-based inhibitory motifs (ITIM) and immunoreceptor tyrosine-based switch motifs (ITSM) and, from that localization site, dephosphorylating and inactivating key proximal signaling molecules of TCR activation. While co-immunoprecipitation of SHP1 and PD1 has been observed in CD4+ T cells and Jurkat T cell lines, suggesting that the two proteins coordinate inhibitory signaling in T cells, more recent data indicates that the activities of SHP1 and PD1 do not entirely overlap [117,118,119]. SHP2 has also been shown to directly interact with PD1 in activated T cells [120]. One possible inhibitory mechanism of SHP2 binding to PD1 is the sequestration of SHP2 away from Lck, from which it removes an inhibitory phosphorylation modification [121].

Targeting SHP1/2 as a means to stimulate immunotherapeutic responses in cancer has been tested [103,104], with somewhat disappointing results. In one phase I study evaluating the PTP inhibitor sodium stibogluconate (SSG) in combination with INFα, no clinical responses were observed in 24 patients with refractory malignancy. A difficulty with small-molecule inhibition of SHPs has been non-specific binding to other phosphatases [122,123,124,125,126], an issue that has been attempted to be addressed by targeting specific allosteric sites of SHP1/2 [122,125,126,127,128]. One allosteric inhibitor of SHP2, SHP099, demonstrated anti-tumor activity and increased immune response in mice inoculated with CT-26 colon carcinoma in a manner that was enhanced by anti-PD1 therapy [129]. This is in contrast to other studies in which T cell-specific deletion of SHP2 did not result in enhanced tumor clearance, or demonstrate the additive potential to anti-PD1 [130]. A possible difference between the studies could be that SHP2 activity in NK cells plays an important role in anti-tumor responses that could not be observed with T cell-specific deletion of SHP2 [131].

SHP1 and SHP2 have been targeted more successfully in preclinical models in therapies that utilize adoptive T cells [132]. Through either genetic deletion of SHP1 or treatment of wild type T cells with shRNA directed against SHP1, adoptively transferred T cells demonstrate enhanced effector function and tumor clearance in the Friend murine leukemia model [132]. Moreover, the introduction of a dominant-negative form of SHP2 in CAR-T cells can impair the inhibitory effect of PD1, and potentially other inhibitory receptors that function through SHP2, on these cells [133]. The enhanced efficacy observed by targeting SHP1 directly in T cells during adoptive therapy versus small-molecule inhibition undoubtedly results from improved specificity against the specific phosphatase and specific cell type of interest. As adoptive T cell strategies mature, coordinate targeting of SHP1 is likely to be of high interest.

### 3.2. PTEN

Phosphatase and Tensin Homolog deleted on Chromosome 10 (PTEN) was first identified as a tumor suppressor gene and is one of the most frequently mutated genes in various cancers [134,135]. PTEN is thought to exert its tumor suppressive function by negatively regulating the PI3K-AKT pathway by dephosphorylating the 3-phosphate group of the lipid substrate phosphatidylinositol-3, 4, 5-triphosphate (PIP3). Proper regulation of PTEN has been found to be of particular importance in maintaining T cell homeostasis. Research in mouse models has shown that loss of PTEN in T cells can lead to the development of T cell lymphomas as well as to the development of autoimmunity, likely because of hyperactive TCR-signaling in the absence of CD28 costimulation [136,137,138,139]. PTEN regulation of T cells is critical for the prevention of T cell lymphoma formation during thymic development of T cells [140], since PTEN loss in mature T cells does not confer enhanced lymphoma risk. PTEN in mature T cells still plays an important role, however, since mice with mature T cells deficient in PTEN develop enlarged spleens, expanded and hyperactivated CD4+ T cells and severe multiorgan autoimmunity [137]. These data suggest that, like SHP1, targeting PTEN will likely be better suited to strategies utilizing adoptive mature T cells to avoid untoward T cell effects resulting from systemic inhibition.

### 3.3. SHIP

SHIP (Src homology 2 (SH2) domain-containing inositol polyphosphate 5-phosphatase) is an additional negative regulator of PI-3-K that inhibits immune cell activity [141]. There are two main isoforms of SHIP with molecular weights of 145kDa (SHIP-1) and 142kDa (SHIP-2). These isoforms function similarly, but differ in their subcellular localization and protein-protein interactions [142]. SHIP-1 is primarily expressed in cells of the hematopoietic lineage while SHIP-2 is ubiquitously expressed. Both enzymes dephosphorylate the 5-phosphate of two substrates–PI(3,4,5)P3 to PI(3,4)P2 and I(1,3,4,5)P4 to I(1,3,4)P3 [143]. The SHIP proteins also contain SH2 domains important for binding to growth factor receptors and immune receptors such as Ly49, CD28, TCRζ, FcRγIIb, and FcγRIII, which facilitate SHIP localization to the plasma membrane where PI(3,4,5)P3 is present [143,144,145].

SHIP has been shown to have phosphatase-dependent as well as phosphatase-independent modes of regulating immune responses [145]. Dephosphorylation of PIP3 by SHIP prevents the binding of effector proteins such as Btk (in B cells) and Vav to PIP3, and additionally inhibits the kinase activity of Akt/PKB [146]. Blocking Vav recruitment to the plasma membrane by PIP3 has been postulated to interfere with Vav-mediated regulation of CD28 costimulation and TCR clustering, although this has not yet been definitively established [26,147]. SHIP also exerts phosphatase-independent functions using its protein-protein interactions. However, most of these studies have not been performed in T cells [148,149,150]. In monocytes, for instance, SHIP disrupts the TLR-4 pathway as well as protein interactions downstream of the NOD2 receptor pathway, thereby leading to a reduction in NF-κB activity [151]. SHIP is likely to be of similar importance to signaling pathways in T cells.

The role of SHIP in immunity has been well studied using various knockout mouse models. Loss of SHIP-1 in T cells induces the differentiation of CD4+ T cells to Th1 and Th17 cells, while reducing differentiation of Tregs [142,151]. SHIP is downregulated in many leukemias and lymphomas, leading to increased levels of Th2 and Tregs and a dampened immune response to lymphoma [144]. SHIP-deficient CD8^+^ T cells show an enhanced cytotoxic response due to the increased expression of the transcription factor, T-bet [152]. Mice with SHIP knockdown in myeloid cells show enhanced anti-inflammatory cytokine response to infection [153,154]. Because of the role of SHIP in limiting Th1 development and CD8^+^ T cell activity, small molecule inhibitors of SHIP are currently being studied as cancer immunotherapies (Table 2) [103,155,156,157].

### 3.4. PEP/PTPN22

PEST-domain enriched tyrosine phosphatase (PEP) is a cytoplasmic phosphatase localized close to the plasma membrane in all hematopoietic cells [158]. PEP physically and tightly interacts with the SH3 domain of the negative regulatory kinase Csk (discussed below), and this complex inhibits T cell activation by opposing phosphorylation of the TCR receptor complex [159,160]. PEP dephosphorylates the Src kinases Lyn and Fyn at their activating phosphorylation sites (Tyr-394 and Tyr-417) in competition with Csk, which phosphorylates the same kinases at their negative regulatory sites (Tyr-505 and Tyr-528) [160,161]. On antigen stimulation, PEP-deficient T cells show increased expression of IL-2 and CD69, as well as increased Erk phosphorylation (during weak TCR complex stimulation) [162]. PEP has also been shown to regulate the activities of Zap-70, TCRζ and Vav1, and the loss of PEP in T cells has been shown to increase T cell association with antigen-presenting cells via increased activation of the small GTPase, Rap1 [163,164].

A single mutation in PEP (C1858T) that reduces PEP’s association with Csk has been associated with over twenty autoimmune diseases including type I diabetes, immune thrombocytopenia, Graves’ disease, lupus and myasthenia gravis [165,166,167,168]. Multiple mouse models have therefore been generated to study the role of PEP in the immune system. While there are some inconsistencies between the studies due to differing genetic backgrounds in the mice models, they universally show that targeting PEP enhances TCR signaling and overexpressing PEP decreases TCR signaling [168]. One of these mouse models uses a PEP C1858T knock-in mutation that results in an amino acid change of R619W in mice corresponding to an autoimmune phenotype generated by R620W in humans [163,168,169]. As with human PEP R620W, PEP R619W in mice demonstrated increased autoimmunity. Although there may exist some subtle difference in biochemical activity between mouse R619W and human R620W (for instance, R620W may have increased intrinsic phosphatase activity), the mouse R619W model has been critical for improved understanding of PEP biological function. Adoptive transfer of PEP-deficient CD8^+^ T cells from mice engineered to express a CD8_+_ TCR specific for ovalbumin (OT-I mice) resulted in enhanced clearance of ovalbumin-expressing EL4 thymoma tumors, and decreased sensitivity to TGFβ [170]. Additionally, there have been multiple successful screening approaches to identify small-molecule inhibitors of this phosphatase [171,172,173,174]. These compounds have the potential to be useful clinically as cancer immunotherapeutics.

### 3.5. PTP-PEST/PTPN12

PTPN12 is a PEST-domain-containing phosphatase that is ubiquitously expressed in mammalian cells [175]. Like PEP, PTPN12 also interacts with Csk, although the functions of the PTPN12-Csk complex are thought to be different from those of the PEP-Csk complex [176]. Because the loss of PTPN12 causes early embryonic lethality in mice, overexpression studies and conditionally targeted knock-down of this phosphatase have been required to study its physiological roles in mature tissues. In B and T cells, PTPN12 functions as a potent negative regulator by interacting with and dephosphorylating key signaling molecules such as Crk-associated substrate (Cas), Shc, Pyk2, Grb2 and Fak, and by inhibiting Ras activation [176,177,178,179,180,181]. PTPN12 also participates in the regulation of actin reorganization and immunological synapse formation in lymphocytes by targeting WASP and Arp2/3. Overexpression of PTPN12 also downregulates the production of IL2 and inhibits NF-κB signaling [179,182,183]. A conditional knock-down of PTPN12 in T cells has identified a role for this protein in secondary (but not primary) T-cell activation, anergy induction, and autoimmunity [179]. Additionally, this phosphatase has been shown to have tumor-suppressive roles by inactivating epidermal growth factor receptor (EGFR) and platelet derived growth factor receptor (PDGFRβ) signaling in breast cancer, and Ras signaling in colorectal cancer [183,184,185,186]. Given its role as a tumor suppressor and its broad distribution, this likely relegates targeting PTPN12 to improving adoptive T cell therapies.

### 3.6. PTPH1

PTPH1 (Protein Tyrosine Phosphatase H1) is a FERM (N-terminal band 4.1-, ezrin-, radixin- and moesin-homology) domain-containing phosphatase has been shown to negatively regulate TCR signaling by dephosphorylating membrane-associated proximal signaling molecules [158,187]. A screen for PTPases that dephosphorylate CD3ζ identified both PTPH1 and SHP1, and PTPH1 was demonstrated to be the predominant PTPase that directly binds and dephosphorylates CD3ζ [99]. However, PTPH1 knockdown mice did not show significant differences in TCR-activation or cytokine production. Further, lipopolysaccharide (LPS)-challenged PTPH1 knockdown mice showed a decrease in MCP-1 and IL10 release indicating that PTPH1 might be dispensable for T cell activation [188,189]. The potential for PTPH1 as a target for immunotherapies has not, to date, been addressed.

### 3.7. PP2A

An additional phosphatase that may play a role in the negative regulation of TCR is the serine/threonine protein phosphatase 2A (PP2A). PP2A is a trimeric complex that contains a scaffolding subunit, a regulatory subunit and a catalytic subunit [190]. The regulatory subunit of PP2A binds to and dephosphorylates Carma1 of the CBM (Carma1-Bcl10-Malt1) complex, thereby leading to reduced NF-κB activation and cytokine production [191,192]. However, PP2A has also been demonstrated to positively regulate T cells by repressing the function of CTLA-4, resulting in increased IL2 production [193]. Experiments using an inhibitor against PP2A have shown that PP2A regulates TCR expression in a concentration-dependent manner. Low concentrations of PP2A inhibitor induce TCR downregulation, whereas high concentration of the inhibitor lead to TCR upregulation [194]. The regulation of T cell specific functions of PP2A will need to better elucidated prior to consideration of this phosphatase as a cancer immunotherapeutic.

### 3.8. Csk

The C-terminal Src kinase (Csk) is a cytoplasmic tyrosine kinase that inhibits proximal T-cell activation. As described earlier, Csk phosphorylates inhibitory tyrosines (Y505 and Y528) within the Src kinases Lck and Fyn. To perform this function, Csk is recruited to the plasma membrane by various scaffolding proteins such as focal adhesion kinase (FAK), the cytosolic PH-domain containing Dok1/2, the adaptor protein TNF receptor-associated factor (TRAF3), and the phosphoprotein associated with glycosphingolipid-enriched microdomains (PAG). The Csk interaction with PAG is the best defined [195,196,197,198,199]. In unstimulated T cells, PAG is a transmembrane protein that localizes to lipid rafts and is usually in a phosphorylated state (the state required for binding to Csk). Upon T cell activation, PAG is dephosphorylated, leading to the release of Csk from the plasma membrane and distance from its potential substrates [200,201,202,203,204]. 

The role of Csk as a negative regulator of T cell activation has been evaluated with overexpression and knockdown studies. Overexpression of Csk suppresses T cell activation while inhibition of Csk results in augmented TCR activation [205,206]. Csk-deficient mice show severe abnormalities in T cell development, although the same is not true for PAG-deficient mice, suggesting that there are redundancies in Csk recruitment to the plasma membrane by other binding partners [207,208,209,210]. Overexpression of Csk has been tested as a potential method to prevent immune overactivation due to off-target binding of TCR-engineered T cells after adoptive transfer [211]. Csk-deficient T cells demonstrate spontaneous proximal TCR activation, although the signaling cascade does not extend distal to PLCγ1 activation without additional signals such as CD28 costimulation [209]. The steady-state TCR signaling activation may preclude targeting Csk as a potential cancer immunotherapeutic.

### 3.9. DGKs

Diacylglycerol kinases (DGKs) are enzymes that phosphorylate the lipid second messenger diacylglycerol (DAG) to form phosphatidic acid (PA) [212]. During T cell activation, activated PLCγ1 cleaves phosphatidylinositol 4,5-bisphosphate (PIP2) in the plasma membrane to form two distinct second messengers, DAG and inositol 1,4,5-triphosphate (IP3) [213]. IP3 activates flux of intracellular Ca^2+^ while DAG activates other proteins involved in TCR activation such as the Ras activating protein RasGRP1 and, in concert with the CD28 costimulatory signal, various isoforms of Protein Kinase C (predominantly θ isoform in T cells) [214,215,216,217]. Flux of intracellular Ca^2+^ signaling activates the transcription factor NFAT, while DAG leads to the activation of the transcription factor AP-1 and, in part, NF-κB. Once activated and translocated in the nucleus, NFAT, AP-1 and NF-κB cooperate to initiate the T-cell activation transcriptional program including genes such as the activation marker CD69 [218,219,220]. Phosphorylation of DAG by DGKs turns off DAG-mediated signaling in T cells resulting in attenuation of T cell function including cytokine production [220]. With respect to tumor biology, tumor infiltrating lymphocytes (TILs) from human tumors have been shown to upregulate DGK isoforms resulting in functional inhibition, and cells from DGK deficient mice show substantial antitumor activity [221,222].

There are multiple isoforms of DGKs, although only two (DGKα and DGKζ) play a role in regulating DAG downstream of the TCR [223]. Both of these isoforms have redundant functions in metabolizing DAG and inducing T cell anergy. However, each have distinctive structural motifs, expression patterns and modes of activation [224,225]. DGKα’s activity and localization to the periphery of the immunological synapse are regulated by the binding of Ca^2+^ and PIP3 to structural motifs specific to this protein (EF-hands and C1 domain, respectively), and by phosphorylation by the Src family kinase, Lck [219,226,227]. Additionally, DGKα demonstrates more pronounced transcriptional downregulation than DGKζ after T cell activation and CD28 costimulation. This has been proposed to occur downstream of PI3K-AKT signaling which blocks FoxO-mediated transcription of DGKα [228]. DGKζ is negatively regulated by the microRNA miR-34a but is positively regulated by PKC-mediated phosphorylation of its MARCKS domain and relocalization to the immunological synapse [229,230]. DGKζ has been found to play a dominant role in DAG metabolism and in the suppression of Ras signaling despite being less abundant in T cells, likely because of its increased kinase activity relative to DGKα in naïve T cells [231,232].

Several groups have studied DGKα and DGKζ knockdown mice to better understand the physiological roles of these proteins. Single knockdown of DGKα or ζ does not affect T cell development or the number of CD4+ or CD8^+^ T cells, but can affect numbers of activated T cells in unchallenged mice [220,233,234]. In contrast, mice deficient in both isoforms of DGK demonstrate a significant reduction in the number of peripheral CD4+ and CD8^+^ T-cells [233]. Pathogen clearance studies have shown that the deficiency of either DGKα or DGKζ enhances immune responses against *Listeria monocytogenes* (LM) infection, but that T cell response were unexpectedly severely impaired in double knockout mice [235]. In contrast, studies from our lab demonstrated that CAR-T cells lacking both DGKα and DGKζ produced a stronger effector response and controlled growth of murine mesothelioma relative to CAR-T cells deficient in either isoform [236]. Similarly, two groups, one using CRISPR/Cas9 (clustered regularly interspaced short palindromic repeats/CRISPR-associated protein 9) mediated deletion of both isoforms in CAR-T cells, and another using a non-specific inhibitor of DGKα (R59022) that can also target the ζ isoform, showed increased control of glioblastoma tumors [237,238]. More specific inhibitors that target only DGKα have been developed over the last few years, although their effect on tumor mouse models is yet to be tested [239,240].

Recently, our lab compared T cell activation and tumor clearance by targeting either Cbl-b or DGKζ in mice [241]. We observed that naïve CD8^+^ T cells from mice lacking either Cbl-b or DGKζ showed enhanced CD8^+^ T cell responses after in vitro stimulation when compared with wild type T cells, an effect that was enhanced with deletion of both genes. However, improved tumor growth control in a model of murine pancreatic adenocarcinoma was observed only in the DGKζ knockout mice and not in Cbl-b knockout mice indicating that DGKζ might be a better therapeutic target. This may be because DGKζ-deficient T cells are significantly resistant to several immunosuppressive factors within the tumor microenvironment such as TGFβ, PGE2 and adenosine, and to other T cell inhibitory pathways such as PD1 [236,238,239,242]. Given these data, we believe that DGKs appear to be promising targets to enhance cancer immunotherapies, either through small molecule inhibition, or through CRISPR-mediated deletion in adoptive T cell therapies. Additional work is needed to determine the potential efficacy of this approach and to evaluate potential induction of autoimmunity.

## 4. Autoimmune Considerations

Any immune checkpoint inhibition therapy carries the risk of induction of autoimmunity. Inhibition of a large majority of the intracellular checkpoints discussed in previous sections such as the Cbl proteins, GRAIL and Deltex1 ligases, as well as phosphatases such as SHP1, PTEN and PTP-PEST, have been shown to predispose mice to autoimmune toxicities [44,58,74,110,137,179,243]. Autoimmunity has also been observed in humans with mutations in intracellular checkpoints discussed in this review including patients with a homozygous mutation in the Itch ligase or mutations in the PEP phosphatase [72,166]. In the case of other negative regulators, while autoimmunity has not been readily observed, the risk of negative consequences of removing brakes from T cells cannot be dismissed. Therefore, each new drug therapy or small molecule inhibitor must be thoroughly evaluated for potential autoimmune side effects. Much effort is currently underway to attenuate autoimmune effects while retaining anti-tumor efficacy. For example, recent work from the Wan lab has shown that cancer vaccine/adoptive cellular therapies can induce type I Interferon signaling-mediated autoimmunity, and that these effects can be attenuated by blocking this IFN release [244]. Significant efforts to identify and test more strategies to reduce the autoimmune side effects of immunotherapy, and more specifically intracellular checkpoint inhibitor treatment, are required to unleash the full potential of CD8^+^ T cells in cancer immunotherapy.

## 5. Conclusions

In this review, we evaluated enzymes that serve as intracellular immune checkpoints in T cells. It is notable that our selection of proteins comprises a fraction of the total number of regulators, including adaptor proteins and other enzymes, that may serve to negatively regulate T cell activation. Additionally, proteins that contribute to negative regulation of T cell activation will undoubtedly be revealed as technologic innovation permits enhanced screening of the T cell genome. For instance, a recent study used a genomic wide Cas9 approach to identify a novel modulator of TCR signaling, Fam49B [245]. In any case, improved understanding of intracellular inhibitory protein tissue distribution, degree of T cell inhibition, propensity for inducing autoimmunity, and efficacy in robust tumor models will be required prior to targeting these proteins in clinical trials (in those proteins in which clinical trials are not already underway). Inhibitory proteins described in this review, and other proteins not yet extensively studied or not yet identified, have the potential to greatly enhance the current state of cancer immunotherapy through modulation of CD8^+^ T cell activity.

## Figures and Tables

**Table 1:** E3 ubiquitin ligase intracellular checkpoints.

Protein	Mechanism of Action	Demonstrated Anti-Tumor Activity	Evidence of Autoimmunity	Development of Small Molecule Inhibitors	Development in Clinical Trials
c-Cbl	-Degradation of critical TCR signaling components (e.g., TCRζ) -Establishment of anergy	No	Yes	−	No
Cbl-b	-Degradation of critical TCR signaling components (e.g., CD3ζ) -Establishment of anergy	Yes	Yes	+	Yes
GRAIL	-Degradation of critical TCR signaling components (e.g., TCRβ) -Establishment of anergy	No, but protein is over-expressed in TILs	Yes	−	No
NEDD4	-Degradation of critical TCR signaling components (e.g., Bcl10)	Yes	No	−	No
Itch	-Degradation of critical TCR signaling components (e.g., Bcl10 and CD3ζ) -Establishment of anergy	No	Yes	++	No
Deltex1	-Substrate undetermined -Establishment of anergy	No	Yes	−	No
TRAF6	-Regulates PI3K-Akt pathway by altering protein localization or function by ubiquitination	Yes [39]	No	+	No
MDM2	-Degradation of NFATc2	Yes	No	+++ (not as cancer immunotherapy)	Yes
Peli1	-Degradation of c-Rel and NIK	No	Yes	−	No
SOCS3	-Substrate undetermined	Yes [40]	No	Yes (in neuron regeneration)	No
SOCS6	-Degradation of Lck	No	No	No	No
Cish	-Degradation of PLCγ1	No	No	No	Yes (NCT03538613)

− None yet developed; −/+ Proposed; + Early development; ++ Validated in pre-clinical trials; +++ In clinical trials.

**Table 2:** Phosphatase/kinase intracellular checkpoints.

Protein (Gene)	Mechanism of Action	Demonstrated Anti-Tumor Activity	Evidence of Autoimmunity	Development of Small Molecule Inhibitors	Development in Clinical Trials
SHP1	-Dephosphorylation and inactivation of critical TCR components (e.g., Zap-70, CD3ζ, and TCRζ)	Yes	Yes	+++	Yes
SHP2	-Sequestered from dephosphorylating and activating Lck by PD1 binding -May facilitate TCR signaling in some instances	Yes (potentially only when inhibited in NK cells)	No	+++	Yes
PTEN	-Inactivation of the PI3K pathway by dephosphorylating PIP3	Yes (when inhibited in mature T cells)	Yes	−	No
SHIP	-Inactivation of the PI3K pathway by dephosphorylating PIP3 and IP4	No	No	+++	Yes
PEP	-Dephosphorylation and inactivation of the critical TCR kinases Lck and Fyn	Yes	Yes	+	No
PTP-PEST	-Dephosphorylation and inactivation of key signaling molecules involved in Ras activation and actin reorganization -Establishment of anergy	No	Yes	−	No
PTPH1	-Dephosphorylation and inactivation of the critical TCR component CD3ζ	No	No	−	No
PP2A	-Dephosphorylation and inactivation of the critical TCR component Carma 1	No	No	+	
CSK	-Phosphorylation and inactivation of the critical TCR kinases Lck and Fyn	No	No	−	No
DGKα	-Phosphorylation and inactivation of the critical TCR component DAG -Establishment of anergy	Yes	No	+ ++	No
DGKζ	-Phosphorylation and inactivation of the critical TCR component DAG -Establishment of anergy	Yes	No	++	No

− None yet developed; −/+ Proposed; + Early development; ++ Validated in pre-clinical trials; +++ In clinical trials.
